# Short-term air pollution exposure decreases lung function: a repeated measures study in healthy adults

**DOI:** 10.1186/s12940-017-0271-z

**Published:** 2017-06-14

**Authors:** Luc Int Panis, Eline B Provost, Bianca Cox, Tijs Louwies, Michelle Laeremans, Arnout Standaert, Evi Dons, Luc Holmstock, Tim Nawrot, Patrick De Boever

**Affiliations:** 1 Flemish Institute for Technological Research (VITO), Boeretang 200, 2400 Mol, Belgium; 2 Transportation Research Institute, Hasselt University, Diepenbeek, Belgium; 3 Centre for Environmental Sciences, Hasselt University, Diepenbeek, Belgium; 4 The Belgian Nuclear Research Centre (SCK●CEN), Mol, Belgium; 5 Department of Public Health, Leuven University (KU Leuven), Leuven, Belgium

**Keywords:** Air pollution, Spirometry, FEV1, Fvc, Pef, Particulate matter, PM10, Ozone, Respiratory health

## Abstract

**Background:**

Daily changes in ambient concentrations of particulate matter, nitrogen oxides and ozone are associated with increased cardiopulmonary morbidity and mortality, with the lungs and their function being a vulnerable target.

**Methods:**

To evaluate the association between daily changes in air pollution and lung function in healthy adults we obtained annual lung function measurements from a routine worker health surveillance program not designed for research purposes. Forced Vital Capacity (FVC), Forced Expiratory Volume in the first second (FEV1), FEV1/FVC and Peak Expiratory flow (PEF) from a cohort of 2449 employees were associated with daily measurements of PM_10_, NO_2_ and ozone at a nearby monitoring station in the North of Belgium. Repeated measures were available for the period 2011–2015.

**Results:**

The mean (SD) PM_10_ concentration on the day of the lung function test was 24.9 (15.5) μg/m^3^. A 10 μg PM_10_/m^3^ increase on the day of the clinical examination was associated with a 18.9 ml lower FVC (95% CI: -27.5 to −10.3, *p* < 0.0001), 12.8 ml lower FEV1 (−19.1 to −6.5; *p* < 0.0001), and a 51.4 ml/s lower PEF (−75.0 to −27.0; *p* < 0.0001). The FEV1/FVC-ratio showed no associations. An increase of 10 μgNO_2_/m^3^ was associated with a reduction in PEF (−66.1 ml/s (−106.6 to −25.6; *p* < 0.001)) on the day of the examination.

**Conclusions:**

We found negative associations between daily variations in ambient air pollution and FVC, FEV1 and PEF in healthy adults.

**Electronic supplementary material:**

The online version of this article (doi:10.1186/s12940-017-0271-z) contains supplementary material, which is available to authorized users.

## Background

Air pollution exposure contributes to all-cause morbidity and mortality. Epidemiological studies indicate that short-term exposure to increased fine particulate matter concentration triggers negative health effects. Inhaled particles can affect the cardiopulmonary system, eventually leading to atherosclerosis, myocardial infarction, stroke, chronic lung diseases, and a wide range of other clinical and subclinical effects [1, 2].

The short-term effects of air pollution exposure on lung function have predominantly been investigated in vulnerable subgroups such as children and asthmatics. Zwozdziak et al. [3] reported small FEV1 reductions in healthy children associated with PM1 and PM2.5 exposure [3]. In a landmark study in London, traffic-related air pollution exposure immediately induced adverse pulmonary effects in adult asthmatics [4]. Walking for 2 h on Oxford Street consistently reduced forced expiratory volume during the first second (FEV1) and forced vital capacity (FVC). Forced expiratory flow was negatively associated with fine particulate matter with a diameter smaller than 2.5 μm (PM_2.5_) and traffic-related nitrogen dioxide (NO_2_) concentrations, whereas FEV1 and FVC were most consistently associated with ultrafine particles and elemental carbon. Since then, observational studies have accumulated evidence about the association between selected outdoor air pollutants and worsening of asthma symptoms [5, 6]. A recent meta-analysis of panel studies in COPD patients revealed that a10 μgPM_10_/m^3^ increase in ambient levels had a small, but statistically significant impact on FEV1 and peak expiratory flow (PEF), but also showed significant heterogeneity across the included studies [7].

Less is known about the acute subclinical effects of air pollution on healthy individuals. Acute lung function reduction was observed after exposure to relatively high levels of PM2.5 and BC in a small panel of traffic police [8]. Rice et al. [9] found effects on FEV1 in relation to short-term exposure to relatively low levels of PM_2.5_, NO_2_ and ozone in 3262 healthy participants in the Framingham Heart Study which disappeared after 48 h. Other experimental studies of acute and personal exposure found no or weak associations with lung function but were underpowered [10–12].

We assessed the associations of selected air pollutants (PM_10_ and NO_2_ and ozone) and lung function in a large retrospective cohort of healthy adult workers, using a repeated measures design. This study adds to the evidence base of respiratory effects caused by air pollution because it was carried out on healthy adults working in a rural area with relatively low concentrations i.e. below the EU limit values, but above the levels proposed by the WHO.

## Methods

### Study design

This retrospective cohort study used data from the occupational medical surveillance program of the Belgian Nuclear Research Centre (SCK●CEN) in Mol (Belgium). All employees of the organizations SCK●CEN, VITO, BELGOPROCESS, BELGONUCLEAIRE and the European School undergo an annual check-up at the SCK●CEN medical centre, including a lung function test based on spirometry. Medical examinations are routinely performed on weekdays in the morning between 8 am and 12 am. We obtained repeated lung function measurements from 2449 adults over a 4-year period between 26/01/2011 and 30/01/2015. The ethics board of University Hospital Antwerp approved the study. All participant information was anonymized and de-identified prior to analysis. No informed consent from the participants was required for the present analysis.

### Lung function

Lung function was measured using a Vitalograph Pneumotrac (Ennis, U.K.) in a standing posture, but otherwise following the most recent guidelines by the European Respiratory Society and the American Thoracic Society with respect to lung function testing in occupational settings [13, 14]. The same device was used throughout the study. Participants were supervised and coached by one of the three trained nurses in this medical surveillance program. Participants performed three respiratory manoeuvres (maximum exhalation) during the clinical visits in the years 2011, 2012 and 2013. Because of the high reproducibility of the measurements, most of the participants performed only two manoeuvres in the years 2014 and 2015. Quality of the manoeuvres and lung function parameters were obtained with the Vitalograph Pneumotrac software. Forced Vital Capacity (FVC), Forced Expiratory Volume in the first second (FEV1), FEV1/FVC ratio and Peak Expiratory Flow (PEF) were obtained.

A measuring session was considered usable when at least two manoeuvres, free from artefacts, passed the test acceptability criteria (quality grade C). A session with a maximum difference between two FEV1 values of 150 mL and a maximum difference between two FVC values of 150 mL was graded as A; when only the former was met, the quality was graded B. The highest volume parameters from usable tests were retained. The manoeuvre having the largest FEV1 + FVC sum was recorded and used for all other parameters in this study. Further information on the use of the Vitalograph can be found in the operation manual provided by the manufacturer.

### Air pollution exposure assessment

Ambient air pollution levels were measured at an official air quality monitoring station of the Flanders Environment Agency (VMM) in Dessel (station number 42 N016), located 6 km east from the SCK●CEN medical centre. This background monitoring station is located near the crossing of two canals that are mainly used for recreation. The monitoring station and the SCK●CEN medical centre are both situated in a rural environment without any major local air pollution sources. The nearest road with car traffic is ~1 km to the south east and the nearest major road (with some heavy vehicles) is about 1.5 km to the east (downwind). Validated daily concentrations for particulate matter with diameter smaller than 10 μm (PM_10_; Thermo Andersen ESM FH 62 I-R), nitrogen dioxide (NO_2_; ThermoFisher Model 42i) and ozone (O_3_; Teledyne API Model T400) were obtained. Air pollution exposure was calculated as the average exposure during the day of each clinical visit (lag 0), the day before (lag 1) or 2 days before (lag 2) the clinical visit. The 24-h mean outdoor temperature and humidity were obtained from the meteorological station Antwerpen-Luchtbal (station number T2 M802, approximately 50 km away from the air quality monitoring station) and used to calculate the apparent temperature with the August-Roche-Magnus approximation.

### Statistical analysis

SAS software (version 9.4, SAS Institute Inc., Cary, NC, USA) was used for database management and statistical analysis. The effect of short-term air pollution exposure on lung function parameters was investigated using the MIXED procedure to account for the clustered data within the same person, i.e. repeated lung function tests. A random intercept model was used and the coefficients and standard errors were estimated under restricted maximum likelihood estimation (REML) with unstructured autocorrelation.

The effects of PM_10_, NO_2_, and O_3_ were evaluated in separate models, with adjustment for an a priori chosen list of covariates including sex, age, age^2^, body mass index (BMI), smoking status (current-former-never), quality grade of the lung function test (A-B-C), apparent temperature, hour of the day, day of the week, month and year of the lung function test. All variables were time-variant except sex. Q-Q plots of the residuals were used to test the assumptions of the model. Estimates are given as ml change in FVC or FEV1, percent change in FEV1/FVC ratio or ml/s change in PEF associated with a 10 μg/m^3^ increase in PM_10_, NO_2_ or O_3_. Analyses for ozone were restricted to the warm season (May to September) when impacts were considered most likely.

Stratification by sex, age, smoking status and influenza season was done to perform secondary analyses. Finally, mixed models that included terms for within- and between-subject air pollution exposure were fitted in addition to the main model because we considered the possibility of differences in between- and within-subject air pollution effects.

## Results

The age of the 2449 participants ranged between 16 and 70 years, with an average of 37 (Inter Quartile Range (IQR) = 18) years when entering the study. The average Body Mass Index (BMI) was 25.4 kg/m^2^ (IQR = 4.8). The study included 1756 (72%) male participants. The majority (94%) was Caucasian. A total of 1739 (71%) participants were non-smokers, 342 (14%) were current smokers, and 368 (15%) reported to be former smokers. For 98% of the sample, the smoking status did not change during the study period. On average, 37% received free influenza immunisation. More details about the study population are given in Table 1. The majority of the participants were office workers with a college degree and similar socio-economic background. A few participants were blue-collar workers. No additional profiling details could be obtained due to the prior anonymization of the dataset.

**Table 1:** Characteristics of the study population at entry of the study (*n* = 2449) and pulmonary outcomes based on all clinical visits (*n* = 5404)

Anthropometrics	
Age, years	37 ± 11
Men, %	72
Ethnicity, %	
Caucasian	94
Other	6
Body Mass Index (BMI), kg/m^2^	25.4 ± 4.0
Smoking status, %	
Never	71
Former	15
Current	14
Pulmonary outcomes	
FVC, L	4.71 ± 1.03
FEV1, L	3.79 ± 0.83
FEV1/FVC, %	80.9 ± 7.5
PEF, L/s	8.78 ± 2.20

Values are percentage or arithmetic mean ± SD. *FVC* Forced Vital Capacity, *FEV1* Forced Expiratory Volume in 1 s, *PEF* Peak Expiratory Flow

A total of 5404 clinical visits with lung function test were obtained for the 2449 participants. Most visits were scheduled on a Monday (22%), Tuesday (26%) or Wednesday (21%). Fewer visits were scheduled on Thursday (16%) or Friday (15%). 49% of the sessions were graded A, 15% B and 36% C, respectively. Most participants (35%) had 3 valid measurements during the study period; 28% had 2 and 25% had a single valid measurement; while 12% had more than 3 valid measurements. The clinical examinations with the lung function tests were on average 435 ± 92 days apart from each other. The FVC ranged from 1.89 to 10.84 L with an average of 4.71 L (IQR = 1.49). The average FVC for males and females was 5.06 and 3.67 L, respectively. FEV1 ranged from 0.9 to 7.35 L with an average of 3.79 L (IQR = 1.18). Additional anthropometric and pulmonary characteristics of the study population are given in Additional file 4: Table S1.

The average concentration of PM_10_, NO_2_ and O_3_ on the day of the clinical visit (lag 0) were 24.9 ± 15.5 μg/m^3^, 23.1 ± 9.6 μg/m^3^ and 44.3 ± 19.0 μg/m^3^, respectively (Table 2). Values for the other exposure lags are given in Table 2. Average annual concentrations of PM_10_ and NO_2_ were well below the European limit values of 40 μg/m^3^ during the entire study period (Additional file 5: Table S2). Daily average concentrations exceeded 50 μg PM_10_/m^3^ on 26, 18, 14 and 13 days in the years 2011, 2012, 2013 and 2014, respectively. This was well below the European regulatory limit of 35 days/year but above the WHO recommended maximum of 3 days/year. Also for ozone, the target value for protection of human health was not exceeded during the study period, i.e. the number of days per calendar year on which the daily maximum 8-h average O_3_ concentration exceeded the level of 120 μg/m^3^ was below 25.

**Table 2:** Exposure characteristics on the day of the lung function test (lag 0), and one and two days before (lag 1 and 2)

Exposure, μg/m^3^	Average ± sd	Min	Q1	Q3	Max	IQR
PM_10_						
Same-day (lag 0)	24.9 ± 15.5	5	15	30	105	15
One day before (lag 1)	24.3 ± 15.6	5	14	29	129	15
Two days before (lag 2)	23.9 ± 15.4	5	14	27	129	13
NO_2_						
Same-day (lag 0)	23.1 ± 9.6	7	16	29	65	13
One day before (lag 1)	21.2 ± 10.0	3	14	27	65	13
Two days before (lag 2)	20.0 ± 10.1	3	12	25	65	13
O_3_ – warm season						
Same-day (lag 0)	44.3 ± 19.0	3	30	58	104	28
One day before (lag 1)	47.0 ± 18.7	3	34	61	104	27
Two days before (lag 2)	46.6 ± 19.4	1	33	60	106	27

Values represent average ± standard deviation (SD), minimum (min), 25th percentile (Q1), 75th percentile (Q3), maximum (max) and interquartile range (IQR) concentrations of particulate matter with diameter < 10 μm (PM_10_), nitrogen dioxide (NO_2_) and ozone (O_3_) during the warm season (May–September)

A 10 μg/m^3^ increase in PM_10_ concentration on the same day (lag 0) as the lung function test was significantly associated with a 18.9 ml lower FVC, a 12.8 ml lower FEV1 and a 51.4 ml/s lower PEF (all *p*-values <0.0001). Comparable significant changes were observed for FVC, FEV1 and PEF in association with PM_10_ exposure 1 day (lag 1) and 2 days (lag 2) before the clinical visit (Table 3). In addition, a 10 μg NO_2_/m^3^ increase was associated with significantly lower PEF values (−66.1 ml/s, −66.0 ml/s and −99 ml/s) for lag 0, lag 1 and lag 2 respectively, as well as with lower FEV1 (−13.8 ml) at lag 0. Similar FEV1 reductions were seen at lag 1 and lag 2.

**Table 3:** Estimated change in lung function parameters associated with a 10 μg/m^3^ increase in air pollutant exposure

	PM_10_		NO_2_		O_3_ – warm season	
	Estimated change (95% CI)	*p*-value	Estimated change (95% CI)	*p*-value	Estimated change (95% CI)	*p*-value
Same-day (lag 0)						
FVC, ml	**−18.9 (−27.5 to − 10.3)**	**<0.0001**	−9.8 (−24.1 to 4.6)	0.18	−7.0 (−16.6 to 15.2)	0.93
FEV1, ml	**−12.8 (−19.1 to − 6.5)**	**<0.0001**	**−13.8 (−24.2 to − 3.5)**	**0.009**	4.1 (−8.0 to 16.2)	0.51
FEV1/FVC, %	0.072 (0.02 to 0.17)	0.14	−0.14 (−0.31 to 0.27)	0.10	0.09 (−0.07 to 0.26)	0.26
PEF, ml/s	**−51.4 (−75.0 to − 27.7)**	**<0.0001**	**−66.1 (−106.6 to − 25.6)**	**0.001**	41.0 (−1.0 to 83.0)	0.06
One day before (lag 1)						
FVC, ml	**−23.0 (−31.4 to − 14.6)**	**<0.0001**	−5.6 (−19.4 to 8.3)	0.43	−14.2 (−29.1 to 0.67)	0.06
FEV1, ml	**−15.8 (−21.9 to − 9.6)**	**<0.0001**	−8.8 (−18.7 to 1.2)	0.08	−9.0 (−20.2 to 2.2)	0.12
FEV1/FVC, %	0.089 (−0.00439 to 0.18)	0.06	−0.07 (−0.23 to 0.094)	0.42	0.073 (−0.08 to 0.23)	0.36
PEF, ml/s	**−56.6 (−79.9 to − 33.4)**	**<0.0001**	**−66.0 (−104.4 to − 27.6)**	**0.0008**	−9.9 (−48.9 to 29.0)	0.62
Two days before (lag 2)						
FVC, ml	**−17.8 (−26.6 to − 9.0)**	**<0.0001**	−3.7 (−16.9 to 9.4)	0.58	−10.9 (−24.8 to 3.0)	0.12
FEV1, ml	**−10.7 (−17.1 to − 4.3)**	**0.001**	−9.7 (−19.0 to −4.0)	0.04	−5.2 (−15.6 to 5.3)	0.33
FEV1/FVC, %	0.12 (0.02 to 0.22)	0.016	−0.10 (−0.26 to 0.05)	0.19	0.10 (−0.05 to 0.25)	0.20
PEF, ml/s	**−36.3 (−60.5 to − 12.1)**	**0.003**	**−99.0 (−135.8 to − 22.1)**	**<0.0001**	−10.9 (−46.9 to 25.1)	0.55

Analyses adjusted for sex, age, age^2^, body mass index, smoking status, quality grade of the lung function test, apparent temperature, hour of the day, day of the week, month and year of the clinical visit. *FVC* Forced Vital Capacity, *FEV1* Forced Expiratory Volume in 1 s, *PEF* Peak Expiratory Flow, *PM*_*10*_ particulate matter with diameter < 10 μm, *NO*_*2*_ nitrogen dioxide, *O*_*3*_ ozone. Warm season: May–September. *P*-values < 0.01 are considered significant and are indicated in bold

FVC and FEV1/FVC were not significantly associated with NO_2_ exposure. None of the lung function outcomes were significantly associated with O_3_. Although some results reach borderline significance, overall results for ozone were inconclusive.

**Fig. 1:**
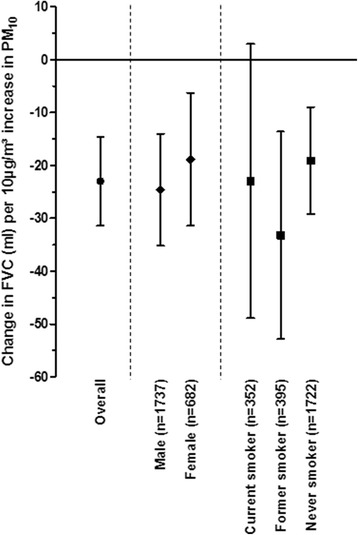
Sensitivity analyses of the association between exposure to particulate matter with diameter < 10 μm (PM10) the day before the clinical visit (lag 1) and Forced Vital Capacity (FVC)

We conducted secondary analyses to investigate the effect of sex, age (older or younger than 30) and smoking status on each of the lung outcome parameters. We did not find evidence for a differential effect of sex or smoking status on our main outcomes, see Fig. 1 for the association between PM_10_ and FVC (all other figures are provided as Additional file 1: Figure S1, Additional file 2: Figure S2 and Additional file 3: Figure S3). Sensitivity analyses excluding people who changed smoking status and excluding measurements made during the influenza season did not affect the conclusions (data not shown).

Models to differentiate between within- and between-subject specific effects were evaluated. Results from these models showed that the reported associations are driven by within-subject effects, which were statistically significant and similar in effect size compared to the results from the main analyses, while the between-subject effects were not significant (data not shown).

## Discussion

This repeated measures study in a population of working adults finds consistent associations between several parameters of lung function and short-term variation in PM_10_ and NO_2_, but not ozone. The observed effect sizes for particulate air pollution are small but they may still have an important public health impact [15]. For instance, in epidemiology they have been shown to be independent indicators of all-cause mortality [16, 17], although spirometry markers alone are not seen as a strict or single criterion to determine lung disease in a clinical setting.

Large cohort studies have mainly looked at the effects of long-term air pollution exposure on lung function. In the Framingham Heart Study and ESCAPE, each 10 μg/m^3^ increase in (long-term) PM_10_ exposure was associated with a reduction in FVC of respectively 65 ml and 59 ml [18, 19]. Assuming that 1 μg/m^3^ PM_2.5_ corresponds to 1.43 μg/m^3^ PM_10_ [20], our short-term associations between FVC and FEV1 and exposure to gaseous pollutants (NO_2_) are smaller than the long-term associations reported by aforementioned studies. However, other studies have also reported smaller effect sizes for acute effects [9, 19]. The fact that short-term effects are smaller than those reported for long-term air pollution exposure might be suggestive for a cumulative effect of air pollution exposure. Similar to the results of the Framingham Heart study cohort, we did not find an association between air pollution and the FEV1/FVC ratio [18, 21].

The fact that we observe an association between lung function and air pollution exposure on the same day is remarkable, but is also known for other impacts. A large recent review found that air pollution increased the risk of stroke significantly on the day of maximum exposure to air pollution, with risk decreasing on following days, demonstrating a clear short-term association [2]. In addition, a multicentre panel study found that short-term increases in particulate matter and ozone can worsen respiratory symptoms of patients with asthma and chronic obstructive pulmonary disease [22].

There are several strengths to underline in this study. We used a repeated measures design in a relatively large dataset from a homogenous population of healthy workers normally not considered to be vulnerable to current levels of air pollution in Belgium. A homogeneous study population reduces between-individual variability and further increases statistical power; the longitudinal study design (within-subject repeated measures) improves statistical efficiency by reducing potential confounding by personal characteristics that do not vary over time. Spirometry measurements were always made with the same instrument and on the same time of day (8 am–10 am), which excludes between instrument and within-day variations in pulmonary function [23, 24]. In addition, the subjects were unaware of the objectives of our the study, which makes participation bias very unlikely. The effects persisted when the analysis was repeated for smaller subgroups such as current and former smokers and women. The effect of air pollution exposure on FVC and FEV1 appeared to be the same in men and women; this is in line with Rice et al. [9] who also found no difference between men and women.

There are, however, several limitations to be mentioned as well. NO_2_ is a traffic-related pollutant that is known to exhibit a large spatial variation in concentration. Because the monitoring station used is situated 6 km from the study centre and many participants travelled by car or bicycle to the examination on the same morning, there is likely more exposure misclassification for this pollutant than for PM_10_, which is more homogeneous over time and space. This may have biased our effect size for NO_2_ towards the null and may explain the difference with the ESCAPE study, which used land use regression models to estimate NO_2_ exposure at each participant’s residential address. Residential addresses were not made available in our study, nor was the travel history prior to the medical examination recorded. In this study, we could not use personal monitoring devices to measure the actual air pollution exposure due to the large cohort size. In the future, automated BC and PM_2.5_ measurements will continue at the air pollution monitoring site as will the lung function measurements. This will increase the power to detect associations with these pollutants, as well as provide the opportunity to determine the relationship between ageing and lung function decline. Another important limitation, is that we were unable to include asthmatic status of the members of the cohort. Asthmatic status and other clinical data were recorded in written medical files, which could not be digitized and anonymized for this study. Another weakness is that the spirometry protocol was modified during the study. In 2011–2013 three manoeuvres were performed, and in 2014–2015 only two, which is a deviation from ERS/ATS guidelines. Spirometry results from the second period may therefore be less accurate than in the first period, but sensitivity analyses suggest that it is unlikely to have affected our main finding.

The results for this apparently healthy group of adults are novel because few panel studies have looked at healthy adults. Routinely collected data from occupational health surveys can be a valuable source of research data, and using it should not be discouraged, as long as its limitations are recognised and appropriately discussed. Results cannot be extrapolated to other more vulnerable groups such as children, elderly or people with chronic conditions that prevent them to work. Finally, we have not looked into possible physiological pathways for the effects observed in this study.

## Conclusions

Short-term variation in particulate air pollution affects lung function in healthy adults in an area with concentrations below the European air quality limit but above the WHO threshold.

## Additional files

Additional file 1: Figure S1. Sensitivity analyses of the association between exposure to particulate matter with diameter < 10 μm (PM10) the day before the clinical visit (lag 1) and Forced Expiratory Volume in 1 s (FEV1). (PNG 12 kb)

Additional file 2: Figure S2. Sensitivity analyses of the association between exposure to particulate matter with diameter < 10 μm (PM10) the day before the clinical visit (lag 1) and FEV1/FVC ratio. (PNG 13 kb)

Additional file 3: Figure S3. Sensitivity analyses of the association between exposure to particulate matter with diameter < 10 μm (PM10) the day before the clinical visit (lag 1) and Peak Expiratory Flow (PEF). (PNG 14 kb)

Additional file 4: Table S1. Detailed Characteristics of the study population at entry of the study (n=2,449) and pulmonary outcomes based on all clinical visits (n=5,404). (DOC 36 kb)

Additional file 5: Table S2. Summary pollutant concentration data per year (Station Dessel 42N016) per year. (DOC 49 kb)

## Abbreviations

BC: Black carbon
BMI: Body mass index
COPD: Chronic obstructive pulmonary disease
ESCAPE: European study of cohorts for air pollution effects
EU: European union
FEF25–75: Forced expiratory flow between 25 and 75% of FVC
FEV1: Forced expiratory volume during the first second
FVC: Forced vital capacity
IQR: Inter quartile range
NO2: Nitrogen dioxide
NOx: Nitrogen oxides
O3: Ozone
PEF: Peak expiratory flow
PM10: Particulate matter with an aerodynamic diameters <10 μm
PM_2.5_: Particulate matter with an aerodynamic diameters <2.5 μm
REML: Restricted maximum likelihood estimation
SCK●CEN: Belgian nuclear research centre
SD: Standard deviation
VMM: Flanders environment agency
WHO: World Health Organisation
