# Supplementary figures and images for: Short-term air pollution exposure decreases lung function: a repeated measures study in healthy adults
Source: Environ Health. 2017 Jun 14;16:60. doi: 10.1186/s12940-017-0271-z (PMC5471732; doi:10.1186/s12940-017-0271-z)

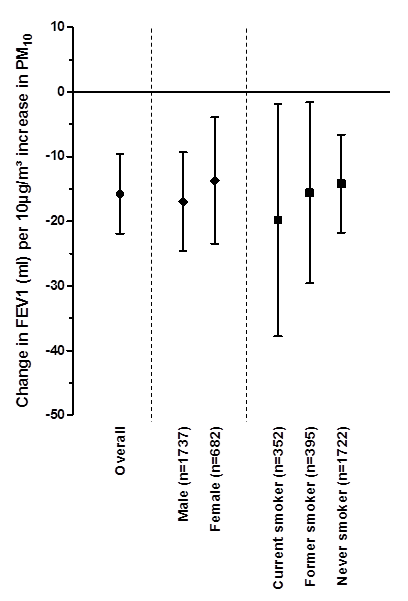

Supplement: Supplementary file 1 — Sensitivity analyses of the association between exposure to particulate matter with diameter < 10 μm (PM10) the day before the clinical visit (lag 1) and Forced Expiratory Volume in 1 s (FEV1). (PNG 12 kb) [file 12940_2017_271_MOESM1_ESM.png]

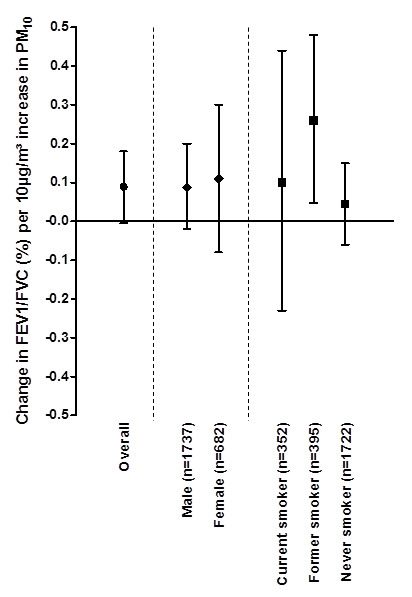

Supplement: Supplementary file 2 — Sensitivity analyses of the association between exposure to particulate matter with diameter < 10 μm (PM10) the day before the clinical visit (lag 1) and FEV1/FVC ratio. (PNG 13 kb) [file 12940_2017_271_MOESM2_ESM.png]

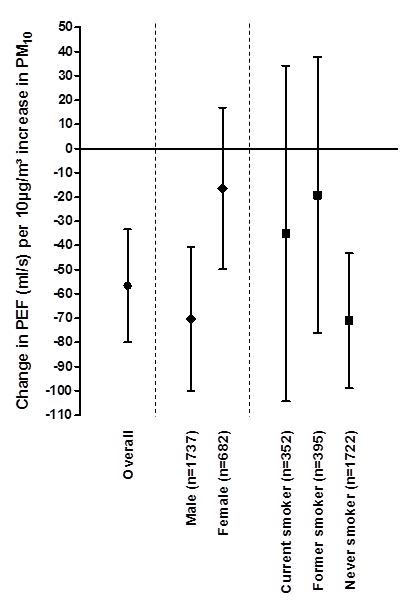

Supplement: Supplementary file 3 — Sensitivity analyses of the association between exposure to particulate matter with diameter < 10 μm (PM10) the day before the clinical visit (lag 1) and Peak Expiratory Flow (PEF). (PNG 14 kb) [file 12940_2017_271_MOESM3_ESM.png]
