# Supplementary material for: Short-term air pollution exposure decreases lung function: a repeated measures study in healthy adults
Source: Environ Health. 2017 Jun 14;16:60. doi: 10.1186/s12940-017-0271-z (PMC5471732; doi:10.1186/s12940-017-0271-z)
Supplement: Supplementary file 4 — Detailed Characteristics of the study population at entry of the study (n=2,449) and pulmonary outcomes based on all clinical visits (n=5,404). (DOC 36 kb) [file 12940_2017_271_MOESM4_ESM.doc]

| **Table** **S1:** Detailed Characteristics of the study population at entry of the study (n=2,449) and pulmonary outcomes based on all clinical visits (n=5,404). | | | | | | |
| --- | --- | --- | --- | --- | --- | --- |
|  | Average ± sd | Min | Q1 | Q3 | Max | IQR |
| **Anthropometrics** |  |  |  |  |  |  |
| Age (years) | 37.29 ± 11.27 | 16 | 28 | 46 | 70 | 18 |
| BMI (kg/m²) | 25.39 ± 4.02 | 16.59 | 22.60 | 27.44 | 54.94 | 4.84 |
| **Pulmonary outcomes** |  |  |  |  |  |  |
| FVC (L) | 4.71 ± 1.03 | 1.89 | 3.93 | 5.42 | 10.84 | 1.49 |
| FEV1 (L) | 3.79 ± 0.83 | 0.90 | 3.19 | 4.37 | 7.35 | 1.18 |
| FEV1/FVC (%) | 80.89 ± 7.54 | 37 | 77 | 85 | 1 | 8 |
| PEF | 8.78 ± 2.20 | 1.45 | 7.10 | 10.33 | 17.13 | 3.23 |
