# Supplementary material for: Short-term air pollution exposure decreases lung function: a repeated measures study in healthy adults
Source: Environ Health. 2017 Jun 14;16:60. doi: 10.1186/s12940-017-0271-z (PMC5471732; doi:10.1186/s12940-017-0271-z)
Supplement: Supplementary file 5 — Summary pollutant concentration data per year (Station Dessel 42N016) per year. (DOC 49 kb) [file 12940_2017_271_MOESM5_ESM.doc]

| **Table** **S2:** Summary pollutant concentration data per year (Station Dessel 42N016) per year. |
| --- |

| Concentration µg/m³ | Average ± sd | Min | Q1 | Q3 | Max | IQR | missing days |
| --- | --- | --- | --- | --- | --- | --- | --- |
| **PM_10_** |  |  |  |  |  |  |  |
| 2011 | 25.5 ± 15.1 | 9 | 15 | 29 | 105 | 14 | 15 |
| 2012 | 22.2 ± 14.2 | 6 | 14 | 26 | 129 | 12 | 8 |
| 2013 | 22.4 ± 12.3 | 5 | 15 | 26 | 83 | 11 | 6 |
| 2014 | 21.4 ± 12.1 | 6 | 13 | 26 | 84 | 13 | 6 |
|  |  |  |  |  |  |  |  |
| **NO_2_** |  |  |  |  |  |  |  |
| 2011 | 20.4 ± 8.7 | 5 | 13 | 26 | 52 | 13 | 39 |
| 2012 | 20.3 ± 10.3 | 4 | 13 | 27 | 65 | 14 | 8 |
| 2013 | 19.9 ± 9.8 | 3 | 13 | 25 | 52 | 12 | 7 |
| 2014 | 18.0 ± 8.0 | 4 | 12 | 23 | 47 | 11 | 4 |
|  |  |  |  |  |  |  |  |
| **O_3_** |  |  |  |  |  |  |  |
| 2011 | 41.2 ± 21.3 | 1 | 27 | 53 | 106 | 26 | 24 |
| 2012 | 40.9 ± 20.9 | 1 | 27 | 55 | 111 | 28 | 16 |
| 2013 | 43.1 ± 21.7 | 1 | 26 | 59 | 123 | 33 | 14 |
| 2014 | 45.1 ± 20.5 | 1 | 31 | 58 | 120 | 27 | 27 |
|  |  |  |  |  |  |  |  |
